# Development and implementation experience of a learning healthcare system for facility based newborn care in low resource settings: The Neotree

**DOI:** 10.1002/lrh2.10310

**Published:** 2022-04-06

**Authors:** Michelle Heys, Erin Kesler, Yali Sassoon, Emma Wilson, Felicity Fitzgerald, Hannah Gannon, Tim Hull‐Bailey, Gwendoline Chimhini, Nushrat Khan, Mario Cortina‐Borja, Deliwe Nkhoma, Tarisai Chiyaka, Alex Stevenson, Caroline Crehan, Msandeni Esther Chiume, Simbarashe Chimhuya, the Neotree Team

**Affiliations:** ^1^ Population, Policy and Practice Research and Teaching Department University College London Great Ormond Street Institute of Child Health London UK; ^2^ Children's Hospital of Philadelphia General, Thoracic, and Fetal Surgery Newborn Intensive Care Unit Philadelphia USA; ^3^ Snowplow Analytics London UK; ^4^ Infection, Immunity and Inflammation Research and Teaching Department University College London Great Ormond Street Institute of Child Health London UK; ^5^ Department of Primary Healthcare Sciences University of Zimbabwe Harare Zimbabwe; ^6^ Parent and Child Health Initiative Lilongwe Malawi; ^7^ Biomedical Research and Training Institute Harare Zimbabwe; ^8^ Mbuya Nehanda Maternity Hospital Harare Zimbabwe; ^9^ Paediatric Department Kamuzu Central Hospital Lilongwe Malawi; ^10^ Maternity Division Sally Mugabe Central Hospital Harare Zimbabwe

**Keywords:** behavioural sciences, global health, health services, neonatal

## Abstract

**Introduction:**

Improving peri‐ and postnatal facility‐based care in low‐resource settings (LRS) could save over 6000 babies' lives per day. Most of the annual 2.4 million neonatal deaths and 2 million stillbirths occur in healthcare facilities in LRS and are preventable through the implementation of cost‐effective, simple, evidence‐based interventions. However, their implementation is challenging in healthcare systems where one in four babies admitted to neonatal units die. In high‐resource settings healthcare systems strengthening is increasingly delivered via learning healthcare systems to optimise care quality, but this approach is rare in LRS.

**Methods:**

Since 2014 we have worked in Bangladesh, Malawi, Zimbabwe, and the UK to co‐develop and pilot the Neotree system: an android application with accompanying data visualisation, linkage, and export. Its low‐cost hardware and state‐of‐the‐art software are used to support healthcare professionals to improve postnatal care at the bedside and to provide insights into population health trends. Here we summarise the formative conceptualisation, development, and preliminary implementation experience of the Neotree.

**Results:**

Data thus far from ~18 000 babies, 400 healthcare professionals in four hospitals (two in Zimbabwe, two in Malawi) show high acceptability, feasibility, usability, and improvements in healthcare professionals' ability to deliver newborn care. The data also highlight gaps in knowledge in newborn care and quality improvement. Implementation has been resilient and informative during external crises, for example, coronavirus disease 2019 (COVID‐19) pandemic. We have demonstrated evidence of improvements in clinical care and use of data for Quality Improvement (QI) projects.

**Conclusion:**

Human‐centred digital development of a QI system for newborn care has demonstrated the potential of a sustainable learning healthcare system to improve newborn care and outcomes in LRS. Pilot implementation evaluation is ongoing in three of the four aforementioned hospitals (two in Zimbabwe and one in Malawi) and a larger scale clinical cost effectiveness trial is planned.

## 1 BACKGROUND AND RATIONALE

Worldwide, 2.4 million children younger than 28 days die every year representing 48% of deaths in children under 5 years^1^ and at least a further 2 million are stillborn.^2^ About 90% of newborn deaths and 98% stillbirths occur in low‐resource settings.^2^, ^3^ Most mothers (~80%) now deliver in health care facilities.^4^ However, this increase in facility‐based deliveries is not associated with expected reductions in maternal and newborn mortality.^4^ Up to 70% of newborn deaths and at least half of stillbirths are avoidable through the consistent implementation of low‐cost evidence‐based interventions.^5^, ^6^ Most recent WHO newborn data indicate coverage, quality, and measurement gaps in newborn care.^7^

Health systems strengthening, along with education and training in newborn care are key to saving newborn lives.^7^, ^8^, ^9^ Previous studies have demonstrated the value of educational interventions for healthcare professionals and kangaroo mother care in decreasing newborn case fatality rates. The potential for e‐health technologies and telemedicine to improve newborn care is increasingly being demonstrated.^10^ These digital formats can provide a user‐friendly interface for the implementation of evidence‐based interventions and guidelines, reliable data systems, digital clinical decision support tools and education in one platform.^11^, ^12^ However, even when strong evidence of effectiveness exists, intervention coverage is often low due to lack of IT‐skills training, human resources and finance,^4^, ^13^ as well as lack of co‐development, government buy‐in and alignment with existing country systems.^14^

### 1.1 Reliable capture of routine healthcare data in low resource settings

The capture of routine health data through Electronic Healthcare Records is key to improving quality of care, efficiency and cost‐effectiveness, and a crucial building block for strong health systems.^7^ Yet, few such Electronic Healthcare Record systems exist in low‐resource settings^15^ and where they do, they have historically been focused on specific disease processes such as HIV. Furthermore, data capture and storage systems are predominantly retrospective and paper‐based, making it inefficient to retrieve and use the data to inform care decisions. Weak health information systems, especially gaps in the provision of reliable, accurate disaggregated and timely data to guide decision making have been highlighted as a significant barrier to equitable and sustained improvements in newborn care.^7^

### 1.2 Digital clinical decision support

Paper‐based clinical guidelines have been used for a long time to optimise care; however, uptake is often variable. Increasingly, digital clinical decision support tools are being developed in high‐resource settings, most often linked to Electronic Health Records. These decision support tools provide healthcare professionals with targeted information for a given patient or situation. Healthcare professionals enter data at the point‐of‐care and those data generate case‐specific advice according to evidence‐based guidelines (commonly known as knowledge‐based systems^16^). Digital knowledge‐based clinical decision support tools have been shown to improve diagnosis and treatment decisions. For example, implementation of a sepsis clinical decision support tool in a US hospital was associated with a 53% reduction in adult sepsis‐related mortality.^17^

Few low resource healthcare systems have adopted knowledge‐based clinical decision support systems at this stage. This is despite most small and sick babies (an estimated 30 million worldwide) seeking facility‐based care within these settings.^18^ Knowledge‐based clinical decision support applications have been trialed in Tanzania and Malawi for the community case management of maternal and childhood health conditions.^19^, ^20^ A digital clinical decision support addressing a narrow range of newborn conditions (Noviguide) linked to education in newborn care (but not to Electronic Health Records) has been piloted in Uganda.^21^ A prototype decision support app is under development in Kenya with pilot data pending.^22^ Digital support tools have huge potential to improve clinical outcomes in these settings. However, they rely on robust data capture systems, and evidence for impact and scale up is lacking. Furthermore, there are many gaps in the evidence with which to create clinical guidelines that are relevant to low‐resource settings.^23^

More recently, non‐knowledge based clinical decision support tools are being developed based on adaptive prediction models, including machine learning methods. Examples of how this approach has been successfully applied in child health in high‐resource settings include Longsdale 2020.^24^ Machine learning has shown improvements in predictive models for assessing the need for critical care and risk of mortality on admission to paediatric intensive care units.^25^ Limiting factors in the use of machine learning to optimise data‐driven predictive clinical models are poor quality of data, limited understanding of the platforms they are delivered into, and deficiencies in their implementation into practice.

### 1.3 Education in newborn care to improve quality of care in low‐resource settings

Education in newborn care is essential to upskill healthcare professionals in delivering evidence‐based practice.^9^ Healthcare providers have cited lack of nursing and medical training in the provision of higher‐level neonatal care as a barrier to providing quality in‐hospital care to newborns.^26^, ^27^ Additional barriers to education in newborn care include high staff turnover and frequent reassignment of staff to different units within the facility. Basic newborn care educational programs have been shown to improve knowledge, competence, and appropriate practices regarding newborns. However, not all basic education packages result in a sustained change in practice^22^ and reliance on paper‐based reporting systems has hampered implementation. Neotree has been developed as a solution through its data capture and constant reinforcement of education messages at the point of care.^28^ Digital approaches to education in newborn care are being explored.^21^, ^22^ However, these have not been combined with, and linked to, electronic health records and clinical decision support systems; and sustained improvements in quality of care have yet to be demonstrated.

### 1.4 Learning healthcare systems

In high income countries, data capture, decision support, education, and continuous learning have been combined to create learning healthcare systems to accelerate health system strengthening and performance. The concept of a learning organisation^29^ was first applied to healthcare systems in the United States (US) in 2007 as a way of leveraging electronic health record data to rapidly develop evidence for daily clinical practice and policy.^30^

In learning healthcare systems, data are collected and collated from multiple sources, for example, Electronic Health Records and patient experience. These data are then analysed and interpreted to create knowledge and evidence, for example, optimising existing evidence based clinical guidelines. This knowledge is then fed back into the healthcare system to improve health care and outcomes through a combination of automated delivery of knowledge, such as, through digital clinical decision support tools and education, quality improvement and implementation science.

Quality improvement aims to systematically improve and monitor the quality of care, for example, feedback via electronic data dashboards. Implementation science aims to understand and reduce gaps between what is known (evidence) and how knowledge is translated into practice (behaviours) through various strategies, such as Audit and Feedback. The Audit and Feedback strategy motivates health professionals to improve their practice by visualizing and highlighting the gap between their own performance and desired performance targets.

Ideally, learning healthcare systems promote an iterative, synergistic cycle of interaction between data, knowledge, and practice delivered on an integrated platform, compatible with local systems and culture resulting in a constant state of quality improvement. Learning healthcare systems also offer the capacity for real‐time learning including quasi‐experimental designs, to greatly increase the ability to generate and test hypotheses in a timely manner. A learning healthcare system can exist at any scale be it facility, national or international. Patients, family, and community engagement and the assurance of high standards of data quality, governance, and accessibility are all central to the delivery of a successful learning healthcare system.

A scoping review of global learning healthcare systems described 68 such systems; the majority of these were in the US, two in the UK and only one in a low‐resource setting, Kenya.^31^ One of the seven recommendations of a recent commission into the future of the UK NHS was to develop the culture, capability, and capacity to become a learning health system.^32^ In the US, the “ImproveCareNow” network is a mature learning health system for child health aimed at improving health outcomes for children and young people with inflammatory bowel disease.^33^ This network has demonstrated improvements in remission rates and growth through standardized data collection, monitoring, evaluation, as well as sustainable and collaborative care.

Despite low‐resource settings have the most to gain from learning health systems, such an approach is uncommon, perhaps due to a lack of knowledge, research, or logistical capacity.^34^ The 2016 WHO framework for improving health facility newborn care highlights the need for actionable information systems.^35^ Delaying the development of learning healthcare systems in low‐resource settings until they can be embedded within fully functioning healthcare systems will only exacerbate existing health inequities.

## 2 THE NEOTREE: DESCRIPTION

Over the last 7 years we have worked with teams in the UK (2013 onwards), Bangladesh 2015), Malawi (2016‐2017 and 2019 onwards) and Zimbabwe (2018 onwards) to co‐develop a learning health system for newborn care in low‐resource settings ‐ the Neotree. We have also conceptualised a similar, linked perinatal learning healthcare system ‐ Mummytree.

Throughout the process, we have used open‐source code and maintained local data ownership.^28^, ^36^ The Neotree system combines an android application with accompanying data visualisation, linkage, and export. Its low‐cost hardware and state‐of‐the‐art software support healthcare professionals to improve postnatal care at the bedside. Neotree is a horizontal intervention ‐ aiming to comprehensively address most common newborn disorders as opposed to focusing on one disease. It has been, and can be, readily adapted to incorporate new disease trends in outbreak situations such coronavirus disease 2019 as (COVID‐19).^37^

### 2.1 Collaborating partners

Neotree has been developed through a network of collaborating institutions. The UK based Neotree team, at University College London works in close collaboration with their Zimbabwean and Malawian institutional partners in all their local sites; in Zimbabwe the Biomedical Research and Training Institute (BRTI) and their Ministry of Health and Child Care Electronic Health Record (EHR) team counterparts, and in Malawi the Parent and Child Health Initiative (PACHI) and the Malawi Ministry of Health. For example, a software developer, funded through the Neotree, works across both the Neotree and the Zimbabwean Ministry of Health EHR team.

### 2.2 Logistics of use

**FIGURE 1:**
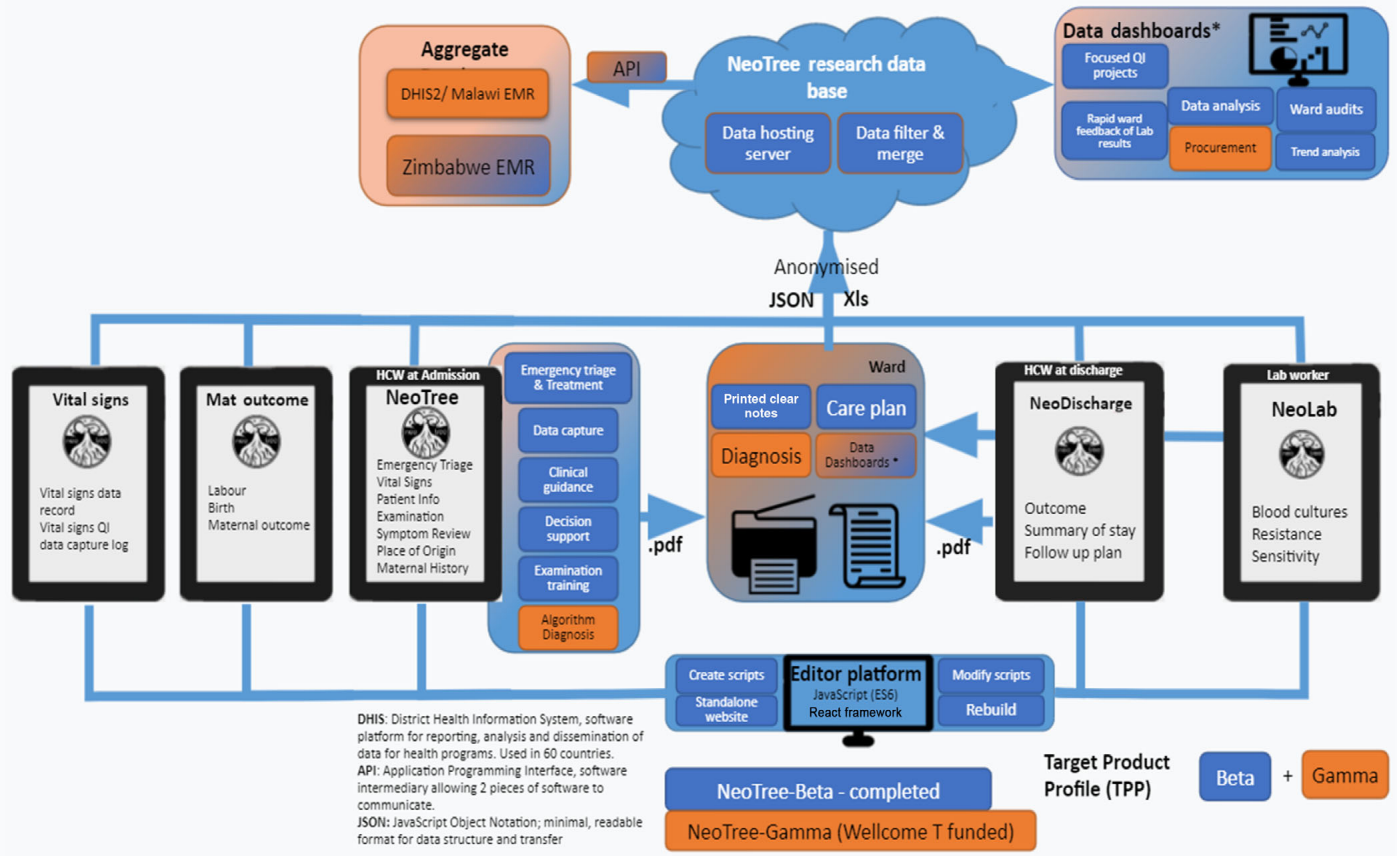
Data pipeline: Currently, the web‐based editor platform is used to design five NeoTree data and decision support forms. HCPs capture data, print to patient notes and export pseudonymized data to a data server. Data are processed, merged and analysed before being presented back to HCPs in meaningful dashboards. Data are also integrated with national electronic medical records

**FIGURE 2:**
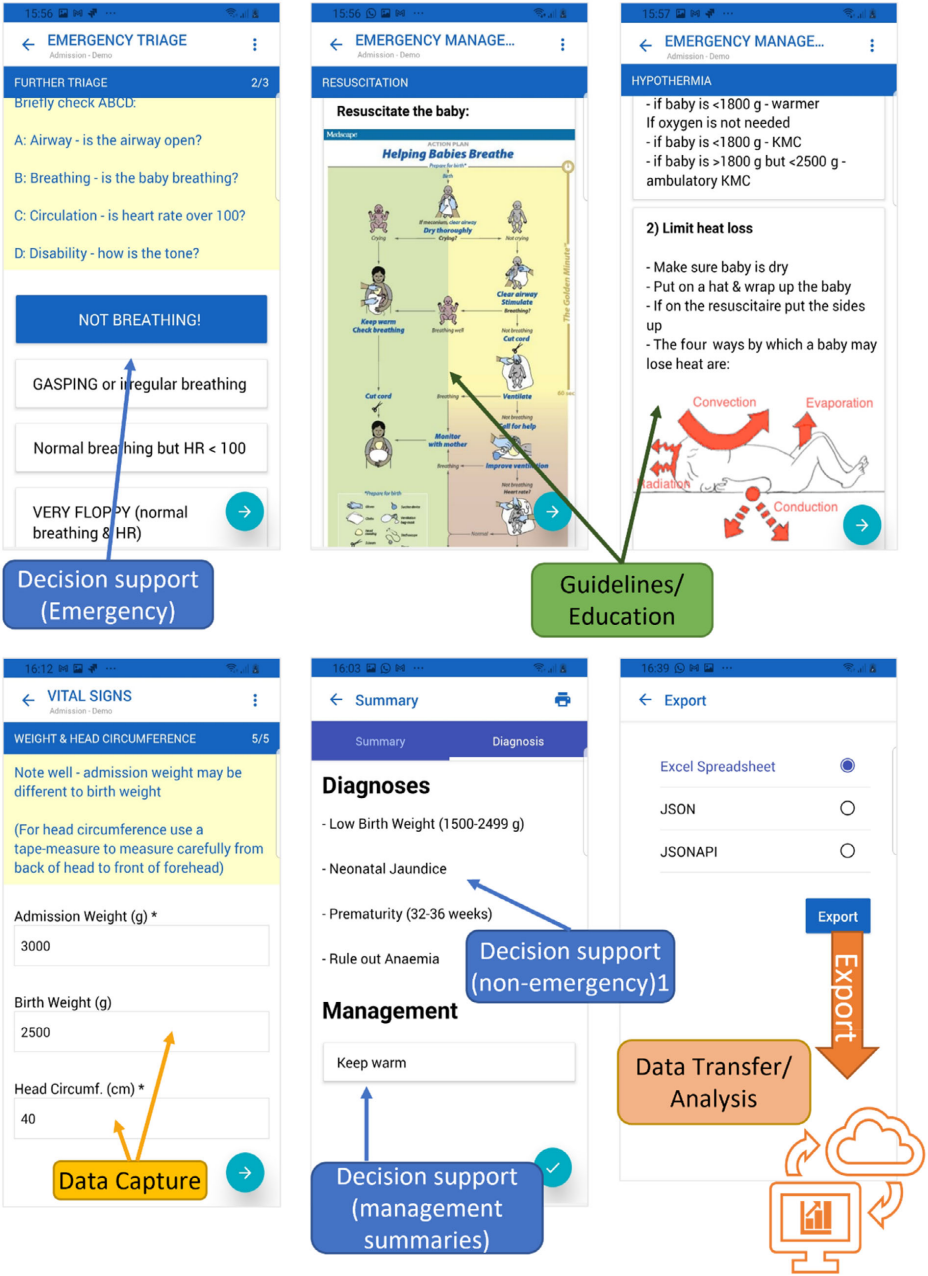
Sample screen shots of NeoTree front end (App interface): Recorded observation, examination, and data capture by the HCP triggers timely guidelines, education, and management giving decision support. On completion, captured data are automatically exported ready for analysis and presentation

Healthcare professionals capture clinical and demographic data on newborns on admission, discharge and from laboratory data using low‐cost android tablets at the bedside (see Figure 1 data pipeline; Figure 2 sample screen shots of the data capture screens). In our country settings (Malawi and Zimbabwe) neonates are usually admitted and discharged by nursing cadres. Hence Neotree has been developed for nurses by nurses. However, in one of our sites (Sally Mugabe Central Hospital) junior doctors take responsibility for admissions and discharges; hence in this site Neotree has been adapted for use by doctors.

### 2.3 Data export and linkage

Neotree has been designed to work in low‐resource settings where network connectivity can be limited Tablets work offline, exporting, and synchronising data when a Wi‐Fi network is available. Data are linked and then fed back to local (hospital) and national (Ministry of Health) audiences (National Electronic Health Record: demo video showing linkage of Neotree to Zimbabwean EHR: https://youtu.be/_hz-hSkpHQI) and aggregate data systems (District Health information software v2).

**FIGURE 3:**
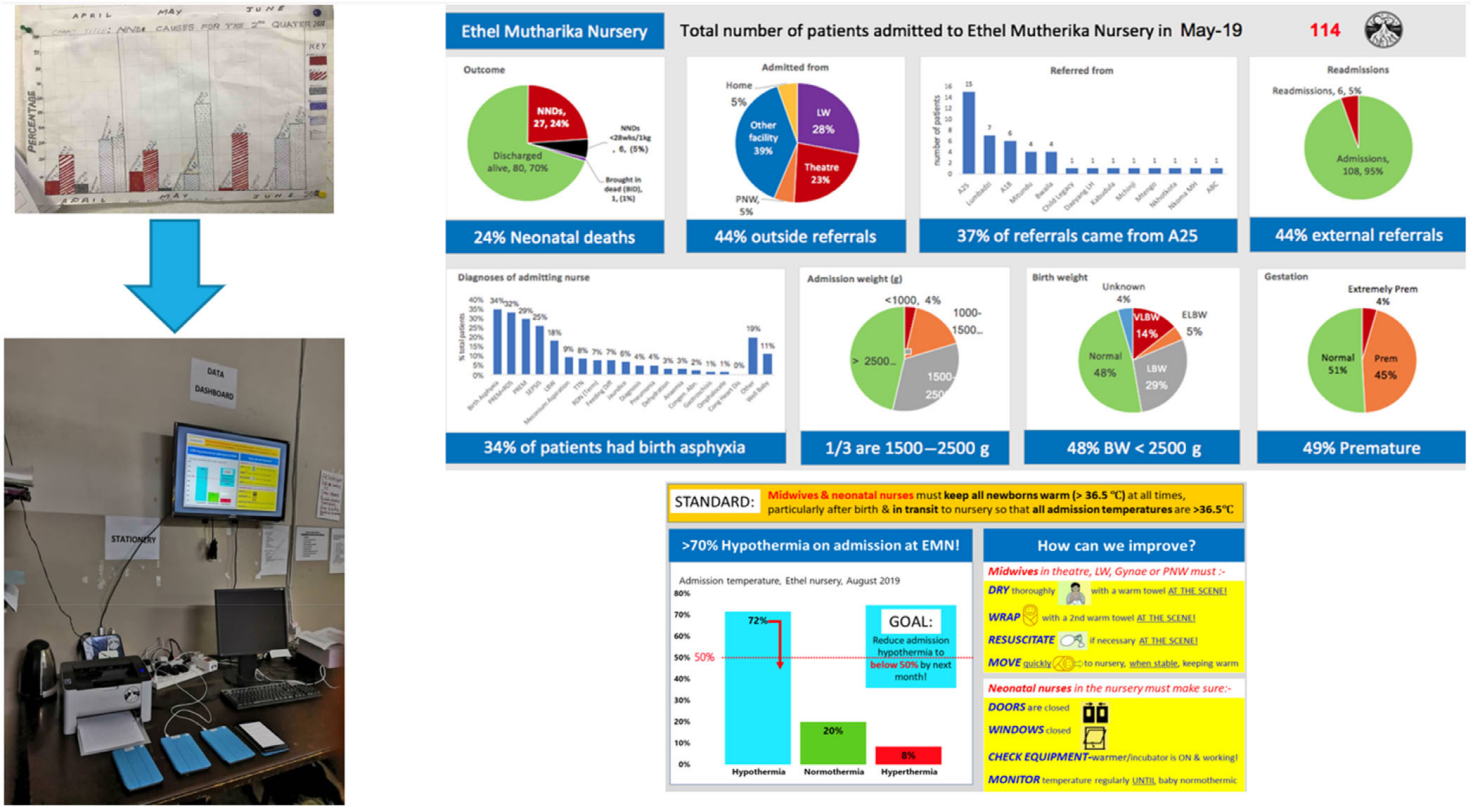
Data dashboard development, Kamuzu Central Hospital, Malawi: Moves units from hand‐drawn, time consuming, unreliable and unclear data charts to clear, dynamic digital displays of meaningful data insights and driving impactful interventions (eg, hypothermia highlighted in lower right dashboard)^38^

Figure 3 shows data visualisations from the Malawian data dashboard prototype co‐developed with Malawian healthcare professionals, incorporating evidence‐based audit and feedback features.^38^

An open‐source development process means the software can be easily adopted by hospitals/governments who can then tailor data input fields locally without needing remote software support or creating commercial dependencies.^36^ In terms of open data curation and management, for example, we work in partnership with the Zimbabwean Ministry of Health and Child Care Electronic Health Records team to ensure seamless integration of Neotree data into the national system, with all data to be held and owned on a Zimbabwean Ministry server.

We are committed to anonymised open data sets being available for in‐country policymakers and clinicians/academics to improve neonatal health outcomes. We follow the FAIR data principles to optimise use and re‐use of our code.^39^ The Neotree and Mummytree have been, and are being, developed to be compatible with local information technology systems and data regulations. This compatibility has been country specific according to country data regulations and preferences. In Zimbabwe, all data are stored on a physical server resident in Zimbabwe. In Malawi, data are stored in an encrypted storage area of the Amazon Web Server. In Zimbabwe, the data export and linkage to the Ministry of Health EHR has been configured precisely to match the national EHR.

Data entry and guidance is tailored to available resources in treatment and technology within the host facility. Data governance procedures have been developed in consultation with both Ministries of Health and the UCL data governance team and are in line with UK General Data Protection Regulations (GDPR). The approach to data security is 2 fold: (i) minimize the chance of data leakage, by ensuring data are encrypted in transit and at rest, and by minimizing the number of places the data are stored (ii) pseudonymizing the data at the point of collection, so that even in the event of a breach, personal identifiable information would not be leaked, and (iii) establishing data backup, storage, and safeguarding procedures.

### 2.4 How the Neotree system aims to improve clinical care

**FIGURE 4:**
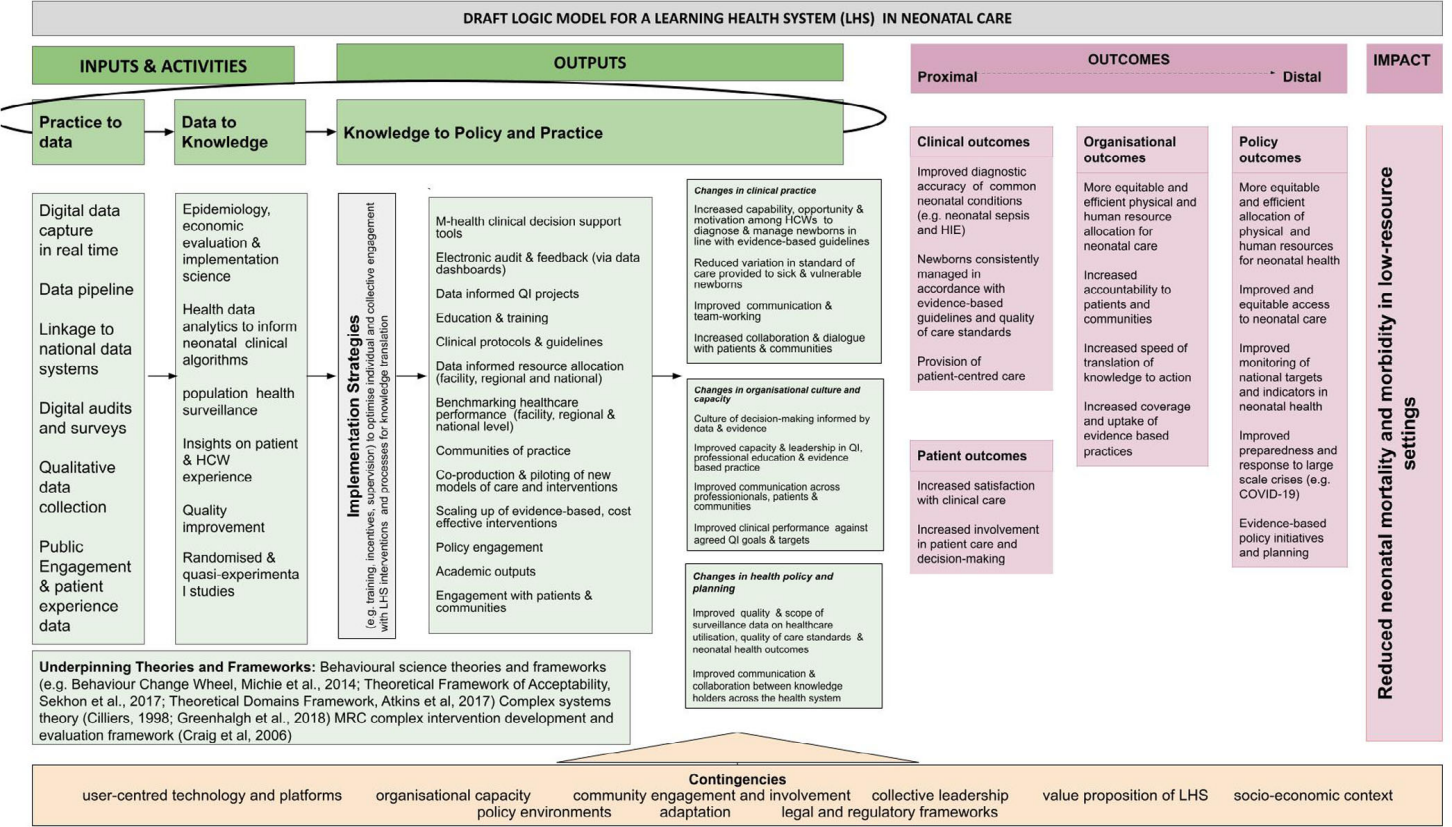
Draft logic model

Knowledge and evidence are generated from the data, for example through the optimisation of existing evidence‐based guidelines for diagnosis and management of sick newborns^23^, ^28^ and clinical surveillance.^37^, ^40^ Generated knowledge and evidence are then used to drive change in clinical practice through four main routes. First, an implementation science driven approach is used to understand relationships between knowledge and behaviours in newborn care in low‐resource settings. Second, evidence‐based clinical decision and management support is provided at the bedside with embedded educational messaging, including dynamic configurability to reflect the needs and capacity of the particular healthcare setting. Third, data are visualised through dashboards (Figure 3) operationalising audit and feedback.^38^ Data dashboards use raspberry pi, a low‐cost computer, linked to screens and an open‐source data visualisation software (Metabase). Currently the dashboard includes two reporting interfaces for the Neotree system. The first dashboard displays real‐time reports on a screen display in neonatal wards visualising key data (eg, admission and mortality rates). The second dashboard is a slide‐deck of monthly data available for presentation at local morbidity and mortality meetings.^41^ A third targeted QI screen for improving key clinical indicators (eg, admission hypothermia) has been prototyped and piloted pending implementation in the future.^38^ The automated delivery of morbidity mortality data to local multi‐disciplinary teams is crucial for clinical decision‐making, resource management, and the monitoring of quality care and standards. However, recent data from the WHO quality of care network show only 17 of 47 countries in the Africa Region undertake these audits.^7^ The final route employed to drive clinical practice is through quality improvement methodology, used by health providers to iteratively improve and monitor care.^42^ Figure 4 shows the draft logic model.

### 2.5 Neotree development

#### 2.5.1 Data capture and interface

A literature review around factors contributing to the stagnation in fall in neonatal mortality in low‐resource settings and possible mitigating strategies informed the Neotree concept development.^43^ A prototype of a data capture, education, and decision support application for tablet devices was developed (2014).^44^ Although this early prototype was not sufficiently functional for field‐testing, the concept and sketches of the user‐interface were found to be acceptable during workshops with Bangladeshi Healthcare professionals (n ~ 15; 2014, unpublished) and conceptualisation was further informed by an audit of six newborn healthcare units in Bangladesh (2014, unpublished).****

In the next phase of development, we iteratively developed Neotree with nurses in a Malawian neonatal unit using agile user‐centred design methods.^28^, ^45^ An editor platform was designed to allow a clinician to configure the application as a digital neonatal admission form (2015‐2016). Hence Neotree‐alpha was developed by digitalising the Malawi‐Ministry of Health neonatal admission paper form within an android application (UK). In 2016 we conducted a qualitative study to explore barriers and enablers to quality newborn care delivery.^26^

A mixed methods pilot evaluation of Neotree‐alpha (2016‐2017) was conducted with 46 Malawian healthcare professionals. Neotree beta MVP1 (Minimum viable product 1) was produced. High acceptability and usability of the data capture of admission information and resuscitation support functions were demonstrated.^28^ Healthcare professionals reported high perceived improvements in their ability to deliver quality newborn care after using Neotree over a one‐month period on the ward. They described improved confidence in clinical decision making, clinical skills, critical thinking, and standardisation of care. Mean systems usability scores (SUS), a quantitative usability measure,^46^ before and after the clinical usability study were high at 80.4 and 86.1 respectively (a mean score of more than 68 indicates good/acceptability usability with only minor changes needed to optimise usability). This compares favourably with other digital systems, for example, a national review of UK Emergency Department Electronic Health Records reported a median SUS of 53^47^ and is similar to a digital clinical decision support tool piloted in Uganda, mean SUS 93.5, n = 12.^21^

In 2018 Neotree‐beta was implemented in Sally Mugabe Central Hospital in Zimbabwe (annual delivery rate ~ 12 000) as a Quality Improvement tool for neonatal sepsis.^42^ Discharge data capture and laboratory data capture functions were added.

Further usability‐focused development o was conducted following implementation in Kamuzu Central Hospital, in Lilongwe, Malawi (annual delivery rate ~ 4500) to create Neotree‐beta MVP‐2.^38^, ^41^, ^48^ A focus of this work was the evaluated co‐development of a dashboard prototype as an electronic data feedback mechanism.^48^ Acceptability of Neotree‐beta MVP2 was found to be high with some feasibility issues raised in an evaluation using behavioural science frameworks.^45^ Throughout this period the data linkage and pipeline features were iteratively developed.

A subsequent Wellcome‐Trust‐funded development and pilot implementation evaluation (funded October 2019 to August 2022) is ongoing in these two hospitals, with a third hospital added in October 2020 (Chinhoyi Provincial Hospital, Zimbabwe, annual delivery rate ~ 3000)^49^ in preparation for wider scale implementation and evaluation. Case fatality rates vary in these units between 190 and 250 per 1000 admitted babies. Since October 2019 upon commencement of Wellcome Trust funding, we have been iteratively improving all existing functions of the Neotree in collaboration with end users and developing additional data collection for stillbirths and maternal outcomes. Clinical, quality of care, process, implementation science and economic data gathered during this time will inform the design and protocol for wider scale evaluation, most likely through a stepped‐wedge trial of clinical and cost effectiveness with embedded process evaluation.

### 2.6 Clinical decision support

The clinical decision support function of the Neotree was developed in three parts. First, national and international standardised neonatal resuscitation and stabilisation guidelines were digitalised and incorporated into Neotree as a resuscitation algorithm, for example, if a baby was not breathing, advice was immediately given on how to resuscitate.^28^ Second, a larger set of clinical decision support algorithms were developed according to Malawian national (Care of the Infant Newborn: COIN^50^) and international guidelines and evidence in newborn care (Table 1). These were configured within the Neotree editor form of the application but not activated as we identified significant gaps in evidence‐based guidance suitable for low‐resource settings. For example, the European definition of neonatal sepsis is two or more clinical symptoms and two or more laboratory signs in the presence of, or because of, suspected or proven infection.^51^ This definition is not possible in low‐resource settings where laboratory investigations are not routinely available. In the absence of extensive trial or epidemiological data in low‐resource settings, alternative techniques to consolidate best available low‐quality evidence can be used, such as expert opinion using the Delphi method.

**TABLE 1:** Clinical decision support algorithms generated by NeoTree (other than resuscitation guidelines) according to degree of complexity (simple vs complex) and level of underlying evidence (strong vs weak)

Data source	Category
1. Simple conditional based decision trees based on based on strong evidence	
Birth weight measured/recorded on admission	Low birth weight
	Very low birth weight
	Extremely low birth weight
	Appropriate for gestational age
	High birth weight
Gestational age recorded on admission	Premature
	Very premature
	Extremely premature
	Term
	Post‐dates
Maternal HIV status recorded on admission	HIV exposed (high risk)
	HIV exposed (low risk)
Maternal Syphilis status recorded on admission	Untreated maternal syphilis
Temperature recorded on admission	Normothermia
	Mild hypothermia
	Moderate hypothermia
	Severe hypothermia
Blood sugar on admission	Hypoglycaemia
	Risk of hypoglycaemia
Abnormal findings on clinical examination	Clinical jaundice
	Clinical convulsions
	Dehydration
	Ambiguous genitalia
	Congenital abnormality
Clinical examination/maternal history	Consider tetanus
2. Simple conditional expressions based on weak evidence	
Abnormal findings on clinical examination/maternal history	Difficulty feeding
	Birth trauma
	Consider abdominal obstruction
	Consider congenital heart disease (CHD)
	Consider anaemia
3. Complex conditional expressions based on strong evidence	
Clinical examination ‐ Thompson score	Neonatal encephalopathy
Clinical examination ‐ triggers/risk factors	Consider Neonatal encephalopathy
Clinical examination/history	Respiratory distress of the newborn^a^
4. Complex conditional expression based on weak evidence (in low resource settings)	
Clinical examination and history (but not investigations)	Risk factors for early onset neonatal sepsis
	Risk factors for late onset neonatal sepsis
	Early onset neonatal sepsis
	Late onset neonatal sepsis
	Consider Necrotising Entero‐Colitis (NEC)
	Consider meningitis

^a^ RDS categories to be developed: Possible meconium aspiration; respiratory distress of prematurity; transient tachypnoea of the newborn; congenital pneumonia; pneumonia/bronchiolitis.

In 2017 to 2018, we conducted a Delphi study to determine whether a panel of 22 neonatal experts with global expertise could address evidence gaps in four neonatal guidelines designed to be included in the Neotree: sepsis, neonatal encephalopathy, respiratory distress, and thermoregulation.^23^ These conditions represent the leading preventable causes of neonatal mortality and are difficult to diagnose and manage appropriately in low‐resource settings with some of the weakest WHO GRADE recommendations and quality of evidence. Key changes made in response to this Delphi study were as follows. First, the Thompson score, a validated sensitive clinical scoring system for diagnosis of neonatal encephalopathy in low‐resource settings,^52^ was adopted. Second, analysis was initiated to identify a set of triggers to prompt the healthcare professionals to carry out a Thompson score assessment, for example, resuscitation longer than 10 minutes after birth.^53^ Third, additional work was commenced to devise and refine a sepsis risk score for low‐resource settings (completion and integration into the Neotree anticipated summer 2022). In 2019, a scoping review of existing literature on clinical prediction models to diagnose neonatal sepsis in low‐resource settings was performed.^54^ In 2020, a dataset was constructed from the routine admission and discharge Neotree data from the neonatal unit of Sally Mugabe Central Hospital, Zimbabwe. A clinical prediction model to diagnose neonatal sepsis was then developed on this dataset by fitting multivariable logistic regression models.^55^ The resulting prediction model is currently being refined on a second training dataset from Zimbabwe. Fourth, all respiratory conditions are being placed under the umbrella diagnosis of respiratory distress of the newborn as experts concluded it was not easy in these settings without access to routine investigations to differentiate between causes of respiratory distress, for example, meconium aspiration vs congenital pneumonia. The remaining non‐resuscitation algorithms are being refined according to best available evidence. All these IF‐THEN knowledge‐based decision algorithms will then be configured via the Neotree editor platform ready for testing.

Finally, clinical management pages were developed during the Zomba Central Hospital^28^ and the Sally Mugabe Central Hospital pilot studies^42^ to encouraging healthcare professionals to take key actions for a given clinical problem. Thus far 31 management pages have been iterated. Currently, the healthcare professional chooses from a list of diagnoses provided to them on the final pages of the Neotree admission pages. After choosing the diagnoses, the associated management pages with advice appear on the Neotree system.

We are currently undertaking usability testing for the automated surfacing of the clinical problem list and linked management pages in response to entered data (rather than by healthcare professional choice). Once non‐resuscitation algorithms have been finalised, we will undertake one‐to‐one usability workshops with exemplar clinical cases followed by implementation evaluation of acceptability and feasibility within the clinical workflow (February to April 2022).

### 2.7 Neotree driving change in clinical care

To date we have gathered data for more than 18 000 babies, and over 400 healthcare practitioners have interacted with the Neotree system. We have observed how the Neotree system can directly and rapidly change clinical care and strengthen adherence to evidence based clinical practice.

An example of such change in clinical care is to manage hypothermia, which is a preventable risk factor for poor outcomes: when the Neotree system was initially rolled out in Kamuzu Central hospital, 79% of babies were admitted hypothermic. This dropped to less than 38% after the data dashboard was deployed and efforts were focused on keeping newborns warm after birth and in transit, prior to admission, but may have correlated with seasonal temperature.

Similarly, when the Neotree system with management guideline support was implemented in Sally Mugabe Central Hospital, Zimbabwe, oral antibiotic prescribing on discharge (not an evidence‐based treatment) fell from 97% to 2%.^42^, ^56^ Inappropriate antibiotic usage is associated with increased antimicrobial resistance.

Through supporting these projects, we recognised clear gaps in training in Quality Improvement methods for healthcare practitioners in low‐resource settings. Neotree deployment has been robust despite external crises (eg, industrial action, economic collapse, and COVID‐19) and data have been used to improve care and monitor healthcare outcomes during COVID‐19.^37^ COVID‐19 clinical management and infection control guidance was incorporated into the Neotree. We have demonstrated the ability to adapt rapidly and respond. For example, in response to clinical evidence of a recent rise in fresh stillbirths (www.bbc.co.uk/news/world-africa-53580559, possibly due to indirect impacts of COVID‐19 on health service provision and use), we mobilised our planned implementation of stillbirth data collection in Zimbabwe. Initial data showed stillbirth rates of 100 to 120 per 1000 births (for comparison, UK rates are 3.3 per 1000).^57^

We have a strong ethos of collaboration. Neotree data from Zimbabwe are being used as part of a national study assessing the impact of the COVID‐19 pandemic on mother‐to ‐child transmission of HIV and syphilis.^58^ In Malawi we have worked closely with the local NEST360 team (nest360.org) on data quality and resource/equipment allocation. We are seeking funding to collaborate with GOAL3 (www.goal3.org) to pilot integration of Neotree with a Bluetooth physiological monitoring system for newborn care in low‐resource settings.

## 3 NEOTREE: KEY INTERVENTION DEVELOPMENT AND IMPLEMENTATION LESSONS

Strong African clinical leadership, Ministry of Health buy‐in and collaboration, user‐centric technology and our flexible approach to development and evaluation have been a key to implementation success. Employing locally based stakeholders has ensured community engagement and has gained essential local expertise and leadership.

Significant overall challenges have included the impact of external crises on clinical care and research activities (COVID‐19 pandemic and industrial action) and balancing the requirements of national digital systems with local clinical quality improvement. Further challenges have included ensuring a common language and understanding across clinical, technology and policy stakeholders and the availability of gold standard diagnostics to support the development and validation of the clinical decision support functionality of the Neotree.

A key lesson learnt from the initial prototype development was the importance of high‐end software to create a functioning application. The initial workshops in Bangladesh led to two changes in intervention development. First, that the application data capture and clinical decision support needed to be adaptable to the clinical setting in terms of medical resources (equipment, medication, staffing seniority and experience and availability of investigations). Second, that the clinical decision support should be horizontal, aiming to address the full range of newborn problems encountered in a low‐resource newborn care unit.

Key technological successes during the initial Neotree‐beta development in Malawi^28^ included the advantages of an intuitive interface between the clinical and research teams and the software code. The interactive editor platform enabled those without software coding skills to configure the data capture forms and clinical decision support without having to alter the base code. Staff turnover is high in these settings and there is currently little or no formal training in newborn care. We learnt therefore, that the educational components needed to be simple but comprehensive, including information on clinical examination as well as differential diagnosis and management. Study participants provided data on implementation strategies which were incorporated into future pilot implementation studies ‐ in particular the use of a paid role for Neotree ambassadors to provide technical support (at between 0.3 and 0.4 full time working equivalent per newborn care unit). Two additional gaps were highlighted ‐ a lack of meaningful parent, family and community engagement and a lack of use of behavioural sciences frameworks with which to optimize intervention development and evaluation. Both gaps have been addressed in subsequent development and evaluation cycles.

Significant challenges were met during the initial pilot implementation study in Sally Mugabe Central Hospital, Zimbabwe, with capturing and linking microbiology laboratory data.^42^ The need to ensure high quality Wi‐Fi for intermittent data sync was identified as a second priority for ongoing implementation.

Key lessons learnt from the initial pilot implementation in Kamuzu Central Hospital (May 2019‐September 2019) work were several fold. First, digital health interventions can be optimised combining both agile user‐focused methodologies with behaviour change frameworks. Second, that data on dashboards should be accompanied by clear messaging around how to act on those data, to motivate change in clinical practice (via audit and feedback). Third, complex data transformations should not be handled within the visualisation programme, but elsewhere in the system (eg, a data pipeline) to reduce network requirements in low resource settings.

### 3.1 Summary and next steps

In this paper we have described our experience of developing and implementing a learning healthcare system for newborn care: Neotree. We are gathering ongoing implementation evaluation data and future steps include collecting robust evidence of clinical and cost effectiveness of impact on newborn care and mortality.

Ensuring adequate data quality and robustness, harmonization and integration with existing infrastructures are some of the key challenges to implementing novel healthcare systems. Therefore, we continue to review our collected data and develop measures to control data quality in the long‐term while ensuring the application is user‐friendly for healthcare professionals without being a burden to productivity.

Over the last year we have also been working with partners to strengthen our parent, family and community engagement with promising results. Work to progress the Mummytree is ongoing, and we are also working with Zimbabwean paediatricians to develop and test a strategy to link Neotree data with neurodevelopmental follow‐up.

The very essence of a learning healthcare system is the ability to continuously learn from data and experience. This adaptive system requires a skilled workforce to support ongoing development and implementation. Therefore, future next steps will also include explicit building of capacity and capability in the clinical and academic workforce in low‐resource settings to enable the sustainable development and delivery of Neotree and other similar systems.

## CONFLICT OF INTEREST

Michelle Heys and Felicity Fitzgerald are both trustees of the Neotree charity (www.neotree.org) but receive no financial payment from this role. Caroline Crehan was a trustee of the Neotree charity (stepped down in 2018) and received no financial payment for this role. There are no other conflicts of interest to declare from any other co‐author.
